# The conducting airways before and after gender‐affirming hormone therapy in transgender individuals

**DOI:** 10.1113/EP093927

**Published:** 2026-07-14

**Authors:** Shalaya Kipp, Andrew H. Ramsook, Alex J. Miller, Samyd S. Bustos, Jorys Martinez‐Jorge, Brian T. Welch, Juan G. Ripoll, Michael J. Joyner

**Affiliations:** ^1^ Department of Anesthesiology and Perioperative Medicine Mayo Clinic Rochester Minnesota USA; ^2^ Department of Kinesiology and Health Sciences University of Waterloo Waterloo Ontario Canada; ^3^ Division of Plastic and Reconstructive Surgery Mayo Clinic Rochester Minnesota USA; ^4^ Department of Radiology Mayo Clinic Rochester Minnesota USA; ^5^ Department of Physiology and Biomedical Engineering Mayo Clinic Rochester Minnesota USA

**Keywords:** feminizing hormone therapy, gender‐affirming hormone therapy, GAHT, masculinizing hormone therapy

## Abstract

On average, females have smaller airways than males. The largest discrepancy in airway size occurs during puberty, when endogenous sex hormone concentrations diverge between males and females. Transgender individuals often use gender‐affirming hormone therapy (GAHT) to align physical characteristics with gender identity. It is unknown whether GAHT influences airway size (luminal area and length). We evaluated the large conducting airways before starting GAHT and while undergoing years of GAHT. This retrospective within‐subject study included 12 adult transgender individuals who underwent CT scanning before starting GAHT and again after ≥1 year of GAHT. For each transgender individual, we identified two matched cisgender controls (one male and one female) who also underwent two CT scans over a comparable time interval for longitudinal comparison. We used a three‐dimensional reconstruction of CT imaging to measure luminal areas. Within‐subject changes in airway size between scans were assessed using Student's paired *t*‐tests in transgender individuals undergoing feminizing hormone therapy. Variance between scans was assessed using Levene's test. Pre‐scans occurred an average of 2.0 ± 3.1 years before beginning GAHT. Transgender persons were on GAHT for an average of 3.3 ± 1.7 years (range, 1.0 – 6.7 years) at their second scan. We found no significant differences in airway luminal cross‐sectional areas for transgender persons on feminizing hormone therapy. The variance in airway size change between scans was not statistically different between transgender individuals and control subjects. Our findings suggest that the size of large conducting airways might remain unchanged after a period (>1 year) on GAHT.

## Abstract (highlights)

- **What is the central question of this study?**
  Does gender‐affirming hormone therapy alter airway luminal area and length in transgender individuals over time?
- **What is the main finding and its importance?**
  In this small, within‐subject cohort, airway size did not appear to change over several years of gender‐affirming hormone therapy. Because airway diameter is a major determinant of airway resistance and the resistive work of breathing, these findings might have implications for clinical care and sports policy.

## 1 INTRODUCTION

Biological sex is an important factor when considering respiratory structure and function (Mann et al., 2021). On average, females have smaller lungs (Bellemare et al., 2003; Torres‐Tamayo et al., 2018) and smaller conducting airways (first to fifth generation) than males (Dominelli et al., 2018; Sheel et al., 2009). Importantly, even when matched for height, females exhibit smaller cross‐sectional areas of the trachea and large conducting airways (Dominelli et al., 2018). Some of the reported sex‐based differences emerge prior to puberty (Becklake & Kauffmann, 1999), with a more pronounced separation in airway size during adolescence (Ripoll et al., 2020), when there is a substantial divergence in endogenous testosterone concentration between males and females (Handelsman, 2017; Senefeld & Hunter, 2024). These pubertal hormonal changes also correspond to the better‐recognized sex‐specific variations in anatomical skeletal traits (height, limb length, etc.), skeletal muscle mass, strength and haematocrit that are observed between adult males and females (Handelsman et al., 2018).

Transgender individuals often use gender‐affirming hormone therapy (GAHT), which includes either masculinizing or feminizing hormone therapy to align their physical characteristics with their gender identity. Transgender persons often experience puberty associated with their biological sex before beginning GAHT later in adulthood. Although information has been emerging about how GAHT might influence physiological variables such as lean body mass, muscle cross‐sectional area, muscle strength and haemoglobin and/or haematocrit (Ford et al., 2022; Harper et al., 2021; Van Caenegem et al., 2015), little is known about the effects of GAHT on pulmonary morphology. Given that airway diameter markedly affects airway resistance (Peters et al., 2021) and, consequently, the resistive work of breathing, understanding whether GAHT changes airway diameter has implications for clinical care and sporting communities. Accordingly, the purpose of this study was to assess airway luminal area and length in transgender individuals before starting GAHT and while undergoing years of GAHT. We hypothesized that the airway luminal area and length would remain relatively unchanged after ≥1 year of GAHT.

## 2 MATERIALS AND METHODS

### 2.1 Ethical approval

This retrospective study was approved by the Institutional Review Board at the Mayo Clinic (IRB #17‐008537) and conformed to the standards of the *Declaration of Helsinki*. All images were originally collected as part of routine clinical care. Informed consent was waived because no identifiers were used, the data already existed, the research did not affect patient care, and the patients did not opt out of their data being used for research.

### 2.2 Cohort selection and eligibility

The Mayo Clinic Transgender and Intersex Specialty Care Clinic provided a comprehensive list of all 670 gender‐affirming surgeries performed (2017–2024). Of these 670 surgeries, 80 were duplicate individuals (more than one surgical procedure). From the remaining 590 individuals, 31 had two or more chest CT scans >1 year apart. CT scans were performed for suspected pulmonary embolisms and other non‐traumatic pulmonary concerns, including evaluation of abnormal imaging findings, lung cancer screening and metastatic disease surveillance. All scans were negative for acute pathology.

Of the 31 individuals with two or more CT scans, we performed a detailed medical chart review to determine if and when they began GAHT. We retained individuals who had a CT scan taken before beginning GAHT and another CT scan after ≥1 year on GAHT. Fourteen individuals matched these criteria; however, two of the individuals had CT scans with poor resolution, where we were not able to calculate luminal area. Thus, our final transgender cohort consisted of 12 individuals (Table 1), 4 receiving masculinizing hormone therapy and 8 receiving feminizing hormone therapy. All transgender persons began GAHT in adulthood.

**TABLE 1:** Characteristic of transgender individuals for both their pre‐scan before starting gender‐affirming hormone therapy (Pre GAHT‐Scan) and while on gender‐affirming hormone therapy (On GAHT‐Scan).

	Pre GAHT‐Scan				On GAHT‐Scan			
Sex recorded at birth	Age (years)	Height (cm)	Weight (kg)	Years before starting GAHT	Age (years)	Weight (kg)	Years on GAHT at time of scan	Hormone therapy and dosage
Female	33	165	75	10.2	44	106	1.2	Testosterone enanthate (Delatestryl) 20–60 mg weekly
Female	31	170	85	0.1	32	92	1.0	Testosterone transdermal (Androderm) 2 mg/24 h patch
Female	34	164	134	0.5	44	119	6.7	Testosterone cypionate (Depo‐Testosterone) 25–30 mg weekly
Female	40	157	83	8.5	54	99	5.4	Testosterone enanthate (Delatestryl) 40–60 mg weekly
Male	42	177	95	1.2	46	99	3.0	Spironolactone (Aldactone) 50–100 mg daily Estradiol (Estrace) 2–4 mg twice daily Progesterone (Prometrium) 100 mg daily
Male	22	170	83	−0.2	27	94	4.4	Spiranolactone (Aldactone) 50 mg twice daily Progesterone (Prometrium) 100 mg daily Estradiol (Estrace) 8 mg daily Estradiol (Vivelle‐Dot) 0.1 mg/24 h patch Estradiol (Climara) 0.1 mg/24 h patch
Male	52	180	97	<0.1	56	100	3.6	Spironolactone (Aldactone) 100 mg daily Estradiol (Climara) 0.1‐0.05 mg/24 h patch
Male	31	190	115	1.2	35	83	3.9	Spironolactone (Aldactone) 50 mg twice daily Estradiol valerate (Delestrogen) 3 mg weekly
Male	67	182	83	0.7	69	79	2.8	Spironolactone (Aldactone) 100 mg daily Estradiol (Estrace) 2 mg twice daily Estradiol valerate (Delestrogen) 3 mg weekly Finasteride (Proscar) 5 mg daily
Male	26	173	109	0.3	28	135	1.8	Spironolactone (Aldactone) 50 mg daily Estradiol (Climara) 0.1 mg/24 h patch Estradiol (Estrace) 2 mg daily
Male	48	173	81	0.8	51	84	2.0	Spironolactone (Aldactone) 50 mg daily Estrodial valerate (Delestrogen) 2 mg weekly Medroxyprogesterone (Provera) 10 mg daily Dutasteride (Avodart) 0.5 mg daily Minoxidil (Loniten) 2.5 mg daily
Male	64	178	87	1.3	69	80	5.0	Spironolactone (Aldactone)100–150 mg daily Estradiol (Climara) 0.06 mg/24 h patch Finasteride (Proscar) 5 mg daily

*Note*: Dosage ranges represent changes in the prescribed dose over the course of therapy. Not all medications were taken concurrently; some were used sequentially as part of an evolving GAHT regimen.

Abbreviation: GAHT, gender‐affirming hormone therapy.

To contextualize the changes seen between scans in our transgender cohort, each transgender individual was matched to two cisgender control individuals (one male and one female), for a total of 24 control individuals. A pool of potential control individuals was obtained using electronic health records through the Mayo Clinic. Control subjects were considered eligible if they had two chest CT scans between 2008 and 2024. CT scans were ordered for suspected pulmonary embolism and were negative in all cases. Control individuals were matched to a transgender individual based on years between scans and age.

### 2.3 Image acquisition

Our institution uses standardized CT algorithms for routine thoracic imaging. A posterior–anterior and lateral topogram is obtained at 120 kV and 35 mA. Spiral acquisitions with a pitch of 1.2 are used. Kilovoltage is set at 120, with a standard milliampere–second value of 140. Images are acquired at end‐inspiration. Post‐imaging reconstructions are obtained in the axial and coronal plane using a B46 kernel. Slice thicknesses of 1.5 and 3 mm are reconstructed. Maximal intensity projections in the axial and coronal planes are completed with a slice thickness of 10 mm and reconstruction increment of 2.5 mm.

### 2.4 Image analysis

Images were analysed using commercially available software (TerraRecon, AQI, Foster City, CA, USA). The software algorithm isolates the airways from other tissue and creates a three‐dimensional reconstruction. Area measurements were taken at three points for each of the following airways: trachea, right main bronchus, intermediate bronchus, right upper lobe, left main bronchus, left upper lobe and left lower lobe (Figure 1). The three points corresponded to the beginning, middle and end of the airway; with the beginning–end set as the points of the beginning of bifurcation. Here, we report the average of the three measures to represent the luminal area for each airway, as previously described (Ripoll et al., 2020; Zaremba et al., 2024). Additionally, the length (distance from beginning to end points) of the trachea, right main bronchus, intermediate bronchus and left main bronchus were assessed.

**FIGURE 1:**
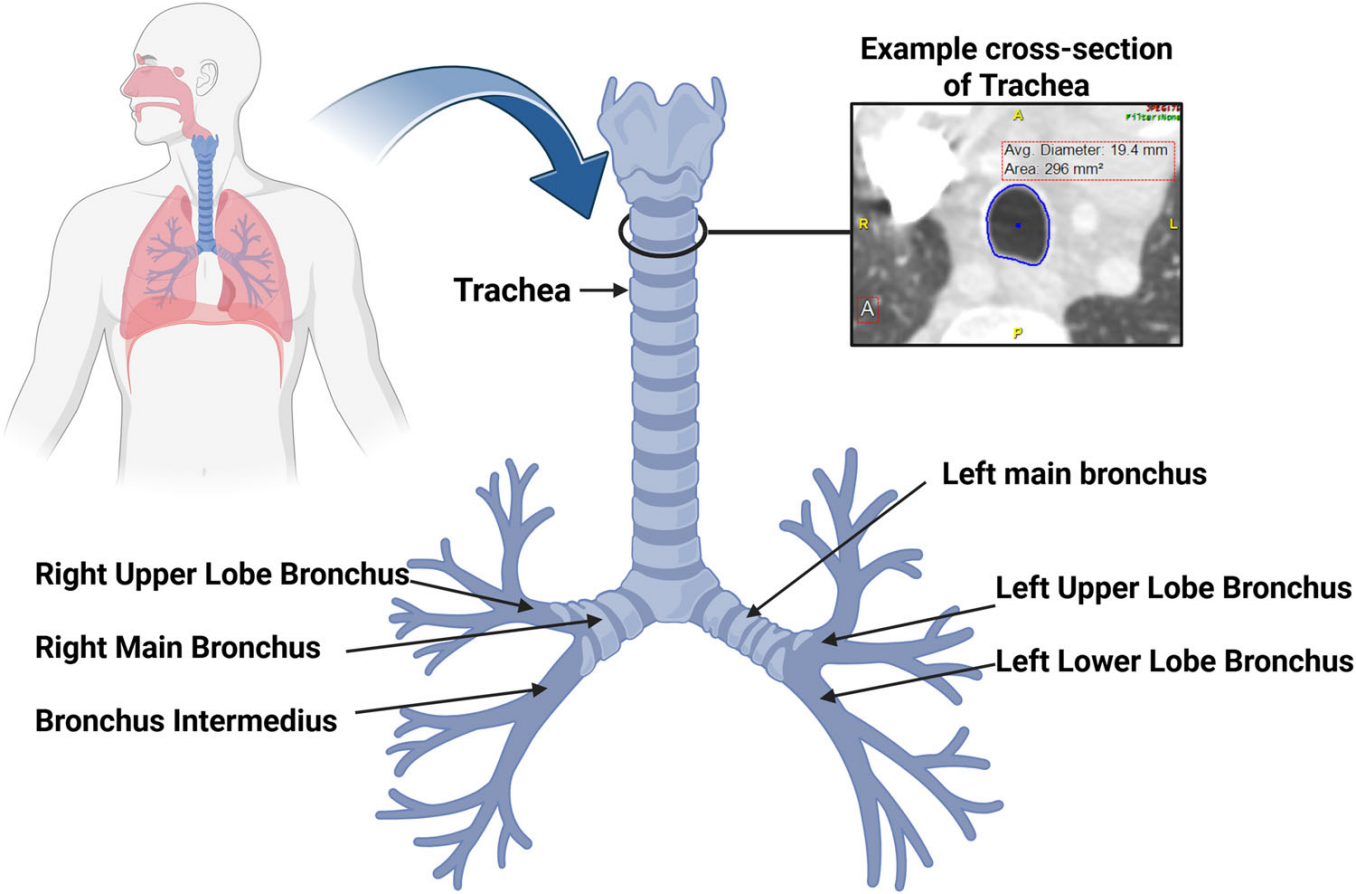
Schematic diagram of airway tree and large conducting airways measured. An example cross‐section of the proximal trachea in Aquarius Intuition (AQi) software used for luminal measurements. Figure created with BioRender.com.

### 2.5 Statistical analyses

Within‐subject changes in airway size between scans were assessed using Student's paired *t*‐tests in transgender individuals on feminizing GAHT. Because the masculinizing hormone therapy group consisted of only four individuals, inferential comparisons were not performed. Normality of residuals was assessed using the Shapiro–Wilk test. To assess variability in airway measurements between scans, the change in airway size between scans was calculated for each individual. Because variability is independent of the direction of change, transgender individuals were pooled across hormone therapy types before comparison with matched cisgender control subjects. Levene's test was used to determine whether variance differed between transgender and control individuals.

Given that there is larger variability in the larger versus smaller airways, we evaluated whether our findings were sensitive to airway segment‐specific variability. We performed an additional analysis using the geometric mean airway lumen diameter across measured airway segments, similar to others (Ross et al., 2024; Smith et al., 2020). We calculated a single summary measure of airway calibre using the geometric mean of available airway lumen diameters across the measured conducting airways. For each airway, the average luminal area $\left(A_{i}\right)$ was converted to an equivalent circular diameter ($D_{i}$) according to Equation 1. The geometric mean airway diameter $\left(D_{\mathrm{GM}}\right)$ was then calculated according to Equation 2, where *n* represents the number of available airway segments for that scan.

(1) $$D_{i}=\sqrt{4A_{i}/\pi A_{i}}$$

(2) $$D_{\mathrm{GM}}={\left(\prod_{i=1}^{n}D_{i}\right)}^{1/n}$$

Analyses were performed using R and RStudio software (R Core Team, 2023; Posit Software, PBC, 2023). Data are reported as the mean ± SD in the tables and text. Given our small sample size, significance was set to *P* < 0.01 to maintain statistical rigour. AI (GPT‐5.5 Thinking, OpenAI) was used to assist with data coding and debugging of R analysis scripts. All AI‐generated suggestions were reviewed, tested, and revised by the authors to ensure accuracy and reproducibility.

## 3 RESULTS

Across the transgender cohort, the pre‐GAHT scans were performed an average of 2.0 ± 3.1 years prior to the transgender individuals starting GAHT. At the time of their second scan, transgender individuals were on GAHT for an average of 3.3 ± 1.7 years, with a range from 1.0 to 6.7 years (Table 1). Control individuals were similar in age to transgender individuals at the time of the first scan (47.3 ± 14.1 vs. 43.8 ± 14.0 years; *P* = 0.264), and the time between scans did not differ between groups (4.9 ± 3.8 vs. 4.8 ± 3.1 years; *P* = 0.325). Participant characteristics were compared using pre‐GAHT values for transgender individuals and first‐scan values for cisgender control subjects. Overall, transgender and cisgender control individuals were similar in height (175.1 ± 7.4 vs. 174.2 ± 13.0 cm; *P* = 0.812), weight (93.7 ± 17.1 vs. 90.1 ± 22.2 kg; *P* = 0.597) and body mass index (30.7 ± 6.3 vs. 29.6 ± 6.5 kg/m^2^; *P* = 0.631). Among transgender individuals receiving feminizing hormone therapy, one was a former smoker, with a smoking history of 22 pack‐years. Among transgender individuals receiving masculinizing GAHT, three were former smokers, with with smoking histories of 11.5, 21.4 and 32.2 pack‐years.

Of the eight transgender individuals receiving feminizing hormone therapy, luminal cross‐sectional area was quantified for all 16 scans of the trachea, right main bronchus, right upper lobe bronchus and left main bronchus; 13 scans of the bronchus intermedius; 12 scans of the left upper lobe bronchus; and 8 scans of the left lower lobe bronchus. Student's paired *t*‐tests were not performed for the left lower lobe bronchus because only four individuals had scans in which luminal area could be quantified. Of the four transgender individuals receiving masculinizing hormone therapy with usable scans, luminal cross‐sectional area was quantified for all eight scans of the trachea, right main bronchus, right upper lobe bronchus, left main bronchus, bronchus intermedius and left upper lobe bronchus; and for six scans of the left lower lobe bronchus.

We found no significant changes to airway luminal area or length following a period of feminizing hormone therapy (all *P*‐values > 0.01; Table 2; Figure 2). The transgender and control individuals showed similar variation in the change in airway size between their two scans compared with the control individuals (all *P*‐values > 0.01; Figure 2). For individuals receiving feminizing hormone therapy, geometric mean airway lumen diameter was not significantly different following GAHT compared with pre‐GAHT values (13.59 ± 0.58 vs. 13.73 ± 0.70 mm; mean change 0.14 ± 0.65 mm; *P* = 0.588). Geometric mean airway lumen diameter was also not significantly different between scans in female control subjects (10.73 ± 0.88 vs. 10.54 ± 0.63 mm; mean change −0.19 ± 0.56 mm; *P* = 0.286) or male control subjects (13.30 ± 1.19 vs. 13.07 ± 1.18 mm; mean change −0.24 ± 0.50 mm; *P* = 0.150).

**TABLE 2:** Airway luminal cross‐sectional areas of transgender persons before and while on gender‐affirming hormone therapy.

	Masculinizing hormone therapy *n* = 4		Feminizing hormone therapy *n* = 8		
Parameter	Before GAHT	On GAHT	Before GAHT	On GAHT	*P*‐value
Airway luminal area					
Trachea, mm^2^	228 ± 48	259 ± 31	321 ± 34	334 ± 44	0.191
Right main bronchus, mm^2^	142 ± 14	160 ± 20	217 ± 31	222 ± 35	0.192
Left main bronchus, mm^2^	97 ± 18	110 ± 18	160 ± 15	168 ± 26	0.228
Bronchus intermedius, mm^2^	85 ± 3	87 ± 13	126 ± 18	135 ± 20	0.350
Right upper lobe bronchus, mm^2^	63 ± 13	68 ± 14	77 ± 13	78 ± 18	0.858
Left upper lobe bronchus, mm^2^	56 ± 11	55 ± 12	102 ± 14	101 ± 18	0.661
Left lower lobe bronchus, mm^2^	44 ± 13	47 ± 17	73 ± 61	75 ± 16	–
Airway length					
Trachea, mm	83 ± 19	90 ± 11	105 ± 13	106 ± 14	0.809
Right main bronchus, mm	15 ± 3	17 ± 3	14 ± 3	15 ± 3	0.410
Left main bronchus, mm	44 ± 3	45± 3	45 ± 4	46 ± 4	0.501
Bronchus intermedius, mm	15 ± 1	14 ± 2	17 ± 4	20 ± 3	0.134

*Note*: Data are presented as the mean ± SD. The *P*‐values correspond to Student's paired *t*‐tests (before vs. on GAHT) in the feminizing hormone therapy group (*n* = 8).

Abbreviation: GAHT, gender‐affirming hormone therapy.

**FIGURE 2:**
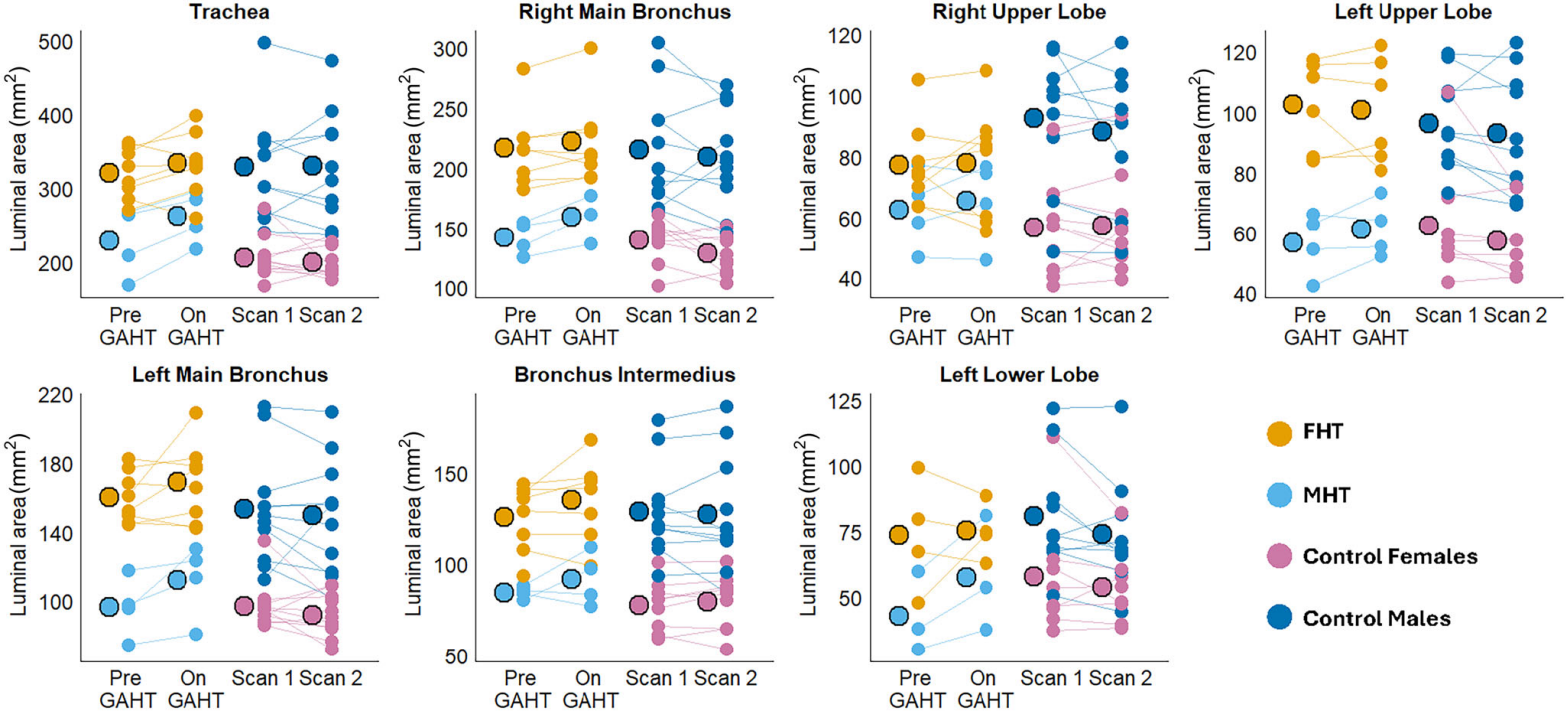
Changes in airway luminal area between scans in transgender individuals and matched control subjects. Transgender individuals are presented according to hormone therapy group: feminizing hormone therapy (FHT; *n* = 8) and masculinizing hormone therapy (MHT; *n* = 4). For transgender individuals, measurements are shown before gender‐affirming hormone therapy (Pre‐GAHT) and while on GAHT. Matched control individuals are presented as control females (*n* = 12) and control males (*n* = 12), with measurements shown at scan 1 and scan 2. Thin lines connect repeated measurements within the same individual. Large outlined circles represent group means. Sample size reflects the total number of individuals in each group; available measurements vary by airway segment.

## 4 DISCUSSION

The primary objective of this study was to evaluate the size (luminal area and length) of the large conducting airways in transgender individuals before starting GAHT, and while undergoing ≥1 year of GAHT. Our major finding was that the size of large conducting airways remains statistically unchanged after a period of GAHT. Additionally, the variation in airway size over time in the transgender individuals was not significantly different from the variation in a group of control individuals who were similar in age and underwent scans approximately the same duration apart as the transgender individuals. This would additionally suggest that small individual changes were probably not attributable to the systematic effect of GAHT.

Our findings indicate that the size of large conducting airways remains unchanged after a period (range 1.0–6.7 years) of feminizing GAHT. Although it might be possible that the time course of airway remodelling takes longer, changes attributable to chronic inflammation seen in airway remodelling happen within weeks to months (Homer & Elias, 2000). Although we found no significant differences in luminal area, it is interesting that almost every airway we measured showed a mean increase (Table 2), regardless of whether the transgender individual was on masculinizing or feminizing GAHT. Prior work has shown an increase in central airway calibre with age in males (Terada et al., 2023), suggesting that ageing might gradually distort and increase the size of the trachea. It is plausible that some of the small mean increases observed in the present study might reflect age‐associated changes in large airway calibre. Additionally, this trend in increasing size might be attributable to small improvements in CT scans and scanners over time, which might have resulted in higher spatial resolution and improved image clarity in the later scans. This small consistent increase is further supported by the fact that the variation in the change inairway size among transgender individuals was not significantly different from that observed in the control group who underwent scans over a similar time span.

Airway dimensions in transgender individuals appeared visually comparable to those of cisgender control subjects matched to sex recorded at birth at both time points. Specifically, individuals undergoing feminizing hormone therapy demonstrated airway sizes that overlapped with control males at both the time points (Figure 2, yellow and dark blue). Likewise, individuals undergoing masculinizing hormone therapy exhibited airway dimensions that overlapped with control females at both comparison points (Figure 2, light blue and pink). Given the limited sample size in this retrospective analysis, formal cross‐sectional statistical comparisons were not performed.

### 4.1 Clinical considerations

In the present study, we only evaluated luminal area of the airways. In clinical practice, pulmonary function testing (PFT) is often used to assess lung function, which is determined by the structure of the airways, in addition to the parenchyma, intrapulmonary vessels and surrounding musculature. Normative PFT data rely on interpretation from data derived from females and males based on sex recorded at birth (i.e. percentage predicted value). European Respiratory Society and American Thoracic Society guidelines suggest that sex recorded at birth yields more accurate predictions, because the effect of GAHT on lung function is poorly understood and the appropriate reference equation for transgender individuals is currently unknown (Graham et al., 2019; Stanojevic et al., 2022). Given that in the present study we did not see any significant changes in luminal area, our results would agree with this conclusion that sex recorded at birth would lead to more accurate predictions of lung function. However, given that the rate of flow an individual can generate is attributable to the radius of the airway and the accompanying skeletal musculature, it is important to consider how GAHT might change the respiratory musculature. Given that testosterone in masculinizing hormone therapy increases lean muscle mass (Ford et al., 2022; Van Caenegem et al., 2015) and oestrogens in feminizing hormone therapy reduce lean muscle mass (Ford et al., 2022; Harper et al., 2021), it is likely that there are longitudinal changes that would influence PFT values. This was demonstrated by Heitzman et al. (2024), who showed increases in maximal expiratory and inspiratory pressures in transgender men undergoing testosterone therapy compared with cisgender females. This highlights the need for transgender‐specific PFT predictive values to assess pulmonary function and standard protocols for working with gender‐expansive populations while conducting PFTs (Tollinche et al., 2021).

In addition to interpretation of pulmonary function, our findings might have implications for airway management in anaesthesia and critical care settings. Because airway luminal dimensions did not change significantly following GAHT, airway anatomy might remain more consistent with sex recorded at birth, particularly among individuals who initiated hormone therapy after puberty (Foer et al., 2021). These observations suggest that clinical decisions involving airway instrumentation, such as endotracheal tube size selection, might continue to be guided by anatomical expectations based on sex recorded at birth rather than gender identity alone. Likewise, preoperative airway risk assessments should recognize that GAHT is unlikely to alter tracheal or bronchial dimensions substantially. Consequently, established difficult airway prediction tools that incorporate sex‐based anatomical differences might remain appropriate for use in transgender patients, while still emphasizing individualized assessment in clinical practice (Cao et al., 2021).

### 4.2 Context for sports and performance

The world of sports has been facing an ongoing challenge to ensure fair and inclusive policies. The primary contention in this ongoing discourse is centred on biological differences between athletes recorded male at birth competing in the female category after GAHT (i.e. transgender women) and females. Biological disparities that profoundly benefit male athletic performance (greater skeletal mass, strength, endurance, cardiac output, haematocrit, etc.) highlight the physiological basis for separate male and female categories in sport (Hunter, 2024; Senefeld & Hunter, 2024). For athletes who start GAHT after puberty, there are some physical characteristics that do not change for both transgender men and transgender women, most notably height and limb length (Hilton & Lundberg, 2021).

Relative to the present study is the natural question, given that gender‐affirming hormone therapy does not change airway size significantly, could transgender women retain a performance advantage with their larger airways? To our knowledge, no studies to date have evaluated the work or cost of breathing in transgender persons. Here, we speculate, based on known sex differences in respiratory anatomy and function. On average, the smaller conducting airways of females directly result in a higher work of breathing during exercise compared with males when matched for ventilation (Peters et al., 2021). A higher work of breathing necessitates a significantly greater rate of oxygen consumption by the respiratory muscles for females compared with males (Dominelli et al., 2015; Kipp et al., 2024). High respiratory muscle work also influences blood flow to the locomotor and respiratory muscles (Dominelli et al., 2017; Harms et al., 1997). Specifically, when the respiratory muscles are unloaded (reducing the work to breathe), there is a greater amount of blood distributed to the primary working locomotive muscles (Harms et al., 1997). Taken together, we speculate that the retention of larger airways by athletes recorded male at birth competing in the female category might be beneficial for exercise performance by reducing the oxygen cost of breathing and allowing more blood to be distributed to the locomotor muscles.

It is important to note that the physiological sex differences discussed (airway size and the resulting effects on respiratory mechanics and cost) reflect average differences between the sexes, with considerable overlap. Thus, not all transgender women will retain the same degree of anatomical advantage. However, in the context of elite sport, where outcomes are often determined by marginal gains, even small differences might have meaningful implications for performance.

### 4.3 Methodological consideration

Chest CT scans provide accurate and reliable data on static luminal area in large conducting airways; however, accuracy diminishes in smaller airway generations (Seneterre et al., 1994), thus we only evaluated the first five generations of airways. Importantly, resistance to flow is higher in the large conducting airways compared with the smaller airway generations (Weibel & Gomez, 1962) and thus plays a greater role in the work of breathing. Furthermore, in the present study we were limited to a smaller sample size of transgender individuals who had two CT scans taken; however, this allowed us to make within‐subject comparisons, which is a strength of our retrospective study. Although future prospective studies are warranted, given the ionizing radiation exposure associated with CT scans, a prospective study might not be ethically feasible. Prospective studies could consider using optical coherence tomography (Peters et al., 2021) to determine whether GAHT is associated with changes in airway structure over time.

An additional consideration of this retrospective analysis is that operational lung volume at the time of CT acquisition was not standardized. Because many scans were obtained in a clinical or emergency setting, participants were instructed to inspire and hold their breath. CT‐derived lung volume was available for both scans in 17 of 34 participants. Among participants with paired lung volume data, CT‐derived lung volume appeared broadly similar between scans; however, because paired lung volume data were incomplete, we were unable to adjust airway calibre measurements statistically for inter‐scan lung volume across the full cohort. Therefore, inter‐scan differences in inspiratory volume remain a potential source of variability in absolute airway area measurements. However, the available paired lung volume data suggest that systematic differences in lung volume are unlikely to explain fully the absence of consistent airway remodelling over time.

Another limitation is the heterogeneity of GAHT regimens among transgender participants, reflecting individualized and evolving treatment plans over time (Table 1). This complexity introduces potential confounders that are difficult to control in a retrospective analysis. Larger cohorts could enable subgroup analyses based on hormone type, dose, duration and delivery method. In addition, changes in body mass index or body composition over time might influence CT image noise and contribute to inter‐scan variability in airway measurements.

## 5 CONCLUSION

In this small cohort, large conducting airway size did not appear to change following a period of feminizing GAHT. Given that airway diameter markedly affects airway resistance and the resistive work of breathing, these findings might have implications for clinical care and sports policy.

## AUTHOR CONTRIBUTIONS

Conceptualization: Shalaya Kipp, Andrew H. Ramsook and Michael J. Joyner. Image and data analysis: Shalaya Kipp. Writing—original draft: Shalaya Kipp and Andrew H. Ramsook. Writing—review and editing: Shalaya Kipp, Andrew H. Ramsook, Alex J. Miller, Samyd S. Bustos, Jorys Martinez‐Jorge, Brian T. Welch, Juan G. Ripoll and Michael J. Joyner. All authors have read and approved the final version of the manuscript and agree to be accountable for all aspects of the work in ensuring that questions related to the accuracy or integrity of any part of the work are appropriately investigated and resolved. All persons designated as authors qualify for authorship, and all those who qualify for authorship are listed.

## CONFLICT OF INTEREST

None declared.

## FUNDING INFORMATION

None.

## GENERATIVE AI STATEMENT

AI (GPT‐5.5 Thinking, OpenAI) was used to assist with data coding and debugging of R analysis scripts. All AI‐generated suggestions were reviewed, tested and revised by the authors to ensure accuracy and reproducibility.

## Data Availability

The datasets generated from the present study are available from the corresponding author on reasonable request.
